# An automated system for rapid cellular extraction from live zebrafish embryos and larvae: Development and application to genotyping

**DOI:** 10.1371/journal.pone.0193180

**Published:** 2018-03-15

**Authors:** Christopher J. Lambert, Briana C. Freshner, Arlen Chung, Tamara J. Stevenson, D. Miranda Bowles, Raheel Samuel, Bruce K. Gale, Joshua L. Bonkowsky

**Affiliations:** 1 Department of Mechanical Engineering, University of Utah, Salt Lake City, Utah, United States of America; 2 Department of Pediatrics, University of Utah School of Medicine, Salt Lake City, Utah, United States of America; 3 Nanonc Inc., Salt Lake City, Utah, United States of America; Hong Kong University of Science and Technology, CHINA

## Abstract

Zebrafish are a valuable model organism in biomedical research. Their rapid development, ability to model human diseases, utility for testing genetic variants identified from next-generation sequencing, amenity to CRISPR mutagenesis, and potential for therapeutic compound screening, has led to their wide-spread adoption in diverse fields of study. However, their power for large-scale screens is limited by the absence of automated genotyping tools for live animals. This constrains potential drug screen options, limits analysis of embryonic and larval phenotypes, and requires raising additional animals to adulthood to ensure obtaining an animal of the desired genotype. Our objective was to develop an automated system that would rapidly obtain cells and DNA from zebrafish embryos and larvae for genotyping, and that would keep the animals alive. We describe the development, testing, and validation of a zebrafish embryonic genotyping device, termed “ZEG” (Zebrafish Embryo Genotyper). Using microfluidic harmonic oscillation of the animal on a roughened glass surface, the ZEG is able to obtain genetic material (cells and DNA) for use in genotyping, from 24 embryos or larvae simultaneously in less than 10 minutes. Loading and unloading of the ZEG is performed manually with a standard pipette tip or transfer pipette. The obtained genetic material is amplified by PCR and can be used for subsequent analysis including sequencing, gel electrophoresis, or high-resolution melt-analysis. Sensitivity of genotyping and survival of animals are both greater than 90%. There are no apparent effects on body morphology, development, or motor behavior tests. In summary, the ZEG device enables rapid genotyping of live zebrafish embryos and larvae, and animals are available for downstream applications, testing, or raising.

## Introduction

Zebrafish (*Danio rerio*) is a small vertebrate model system widely used by the biomedical research community. Zebrafish have rapid development, have transparent embryos, are inexpensive, can generate large numbers of offspring, and have a large variety of molecular and imaging tools available. The zebrafish body plan, organs, and genome are conserved with other vertebrates including in particular humans [1]. Recent work demonstrates that zebrafish can be used for drug discovery in human diseases [2], and for understanding the pathogenicity of mutations discovered by next-generating sequencing approaches in patients [3]. Despite the widespread use of zebrafish, automated research tools for working with zebrafish embryos have not developed at the same pace as the research methodologies. There is a bottleneck requiring skilled labor, which in turn has led to limitations on drug and mutant screens, and an inability to capitalize on the potential for identifying new therapies or to interrogate chemical-genetic pathways.

A zebrafish screen for mutagenesis or for identifying transgenic offspring can involve time- and labor-intensive genotyping of hundreds to thousands of zebrafish. Further, the rapidly expanded use of CRISPR technologies for mutagenesis and knock-in also could be facilitated by rapid genotyping of live embryos. Currently for genotyping, embryos are grown to adult age (two to three months) before manual fin clipping. Fin clipping requires a trained technician four to six hours to prepare cells and genotype 96 fish; as well as the effort and expenses of raising more adult fish than may be ultimately needed.

Alternatively, zebrafish embryos or larvae can be sacrificed and genotyped. If individual animals need to be distinctly genotyped this is even more laborious, and obviously additional testing or use of the animals is not possible. There are a few options that are laborious and slow for manual genotyping of live embryos and larvae [4, 5], but these are impractical on a larger scale. Ideally, technology to rapidly genotype zebrafish embryos (24–72 hours post-fertilization (hpf)) without harming the fish would facilitate current screens, and could lead to future applications that are not feasible considerations currently. While a variety of microfluidic-based approaches have been reported for sorting, visualizing, or monitoring zebrafish [6, 7], they have not been used for genotyping.

We describe our development and optimization of an automated high-throughput device that can genotype live zebrafish embryos and larvae. Based on previous promising proof-of-concept techniques using microfluidic-based fin-clipping or chorionic fluid genetic analyses for genotyping that we developed [8], we now tested and refined a device to generate cells and usable DNA for genotyping, including for analyses by PCR, agarose gel electrophoresis, HRMA (high resolution melt analysis), and sequencing.

## Materials and methods

### Ethics statement

Zebrafish experiments including this work were performed in accordance of guidelines from the University of Utah Institutional Animal Care and Use Committee (IACUC), regulated under federal law (the Animal Welfare Act and Public Health Services Regulation Act) by the U.S. Department of Agriculture and the Office of Laboratory Animal Welfare at NIH, and accredited by the Association for Assessment and Accreditation of Laboratory Care International. The University of Utah IACUC specifically approved this study.

### Fish stocks and embryo raising

Adult fish were bred according to standard methods; embryos were raised at 28.5°C in E3 embryo medium and staged by time and morphology [9].

Fish lines used in this paper were the following: Tg(*myl7*:*EGFP; foxP2-enhancerA*.*2*:*Gal4-VP16*_*413-470*_)^zc72^ [10]; *abcd1*^*sa509*^, and *abcd1*^*zc90*^ [11]. Lines are available through the Zebrafish International Resource Center (ZIRC) or upon request.

### PCR and HRMA

PCR and high-resolution melt analysis (HRMA) was used for genotyping following published conditions [12] with LightScanner Master Mix (Biofire Defense, Inc.) with the following modification: 5 μL of the fluid collected from the Zebrafish Embryo Genotyper (ZEG) (from 11 μL total volume) was used in an 11 μL volume PCR. We used previously reported primers and conditions for PCR of the *sa509* and *zc90* alleles [11]. For Gal4-VP16, we used primers Gal4F3 5’-CGCTACTCTCCCAAAACCAA-3’ and Gal4R3 5’-CTCTTCCGATGATGATGTCG-3’.

For quantitative PCR of genomic DNA from the chip extraction, we used two different genomic samples of known concentration to generate standard curves by 10-fold serial dilutions. PCR was performed using the primers for the *zc90* allele (79 bp amplicon; zc90-12F 5’-GTGGCTCATCTGTATTCAAACCT-3’ and zc90-12R 5’-CAGCCGTTTTAATGAGCGTGTA-3’); or for the gene *bloc1s1* spanning exon 3 to the 3' UTR (296 bp; BLOC1S1-ISHF 5’-GAAATCGGAGACGTGGAGAA-3’, BLOC1S1-ISHR 5’-TGCAACAATTATGGCACTTA-3’). Temperature cycling was as follows: 50°C 2', 95°C 5', then 40 cycles of 95°C 15"– 60°C 15"– 72°C 15", and a dissociation stage 95°C 15"– 60°C 15"– 95°C 15". PCR was performed using Power Up SYBR Green Master Mix (ThermoFisher) on an ABI 7900HT qPCR machine. Ct values were calculated using ABI software (version SDS2.4).

### Cell counts

Immediately after 10 μL samples were collected from the ZEG, 10 μL of Trypan Blue and 2.2 μL of 5 mg/mL diamidino-2-phenylindole (DAPI) were added. After 30 minutes of incubation, 10 μL of sample solution was added to one side of a hemocytometer and examined under a compound microscope. Cell counts was performed by noting the presence of Trypan Blue and DAPI.

### Survival and morphology analysis

Multiple separate experimental replicates (> 10) were performed with n > 25 animals. Survival and morphology was examined under a dissecting microscope with the examiner blinded to the experimental status of the animals.

### Behavior analysis

Larval behavior analysis was performed on 7 dpf (days post-fertilization) larvae in 96-well square bottom plates (Krackeler Scientific) using a video analysis software program (Noldus EthoVision); all videos were recorded at a frame rate of 25 frames per second. Results were analyzed in RStudio (version 1.0.143). Zebrafish larvae were placed into the 96-well plates at 3 dpf; either after being run through the chip, or randomly selected as controls. Spontaneous swimming behavior was recorded as 10.5 minutes of undisturbed swimming with the light on. Light evoked responses were captured in a 5-minute paradigm of 1 minute with the light on and 4 minutes with the light off. Tap response was gathered with a 1-minute recording of swimming with a tap startle at the beginning.

### Statistical analysis

Statistical analyses were performed using Prism6 software (GraphPad). Student’s *t*-test was used for two-way comparisons; comparisons between three or more groups were performed with ANOVA with post-hoc Tukey’s HSD between individual means.

### Chip construction

**Fig 1:**
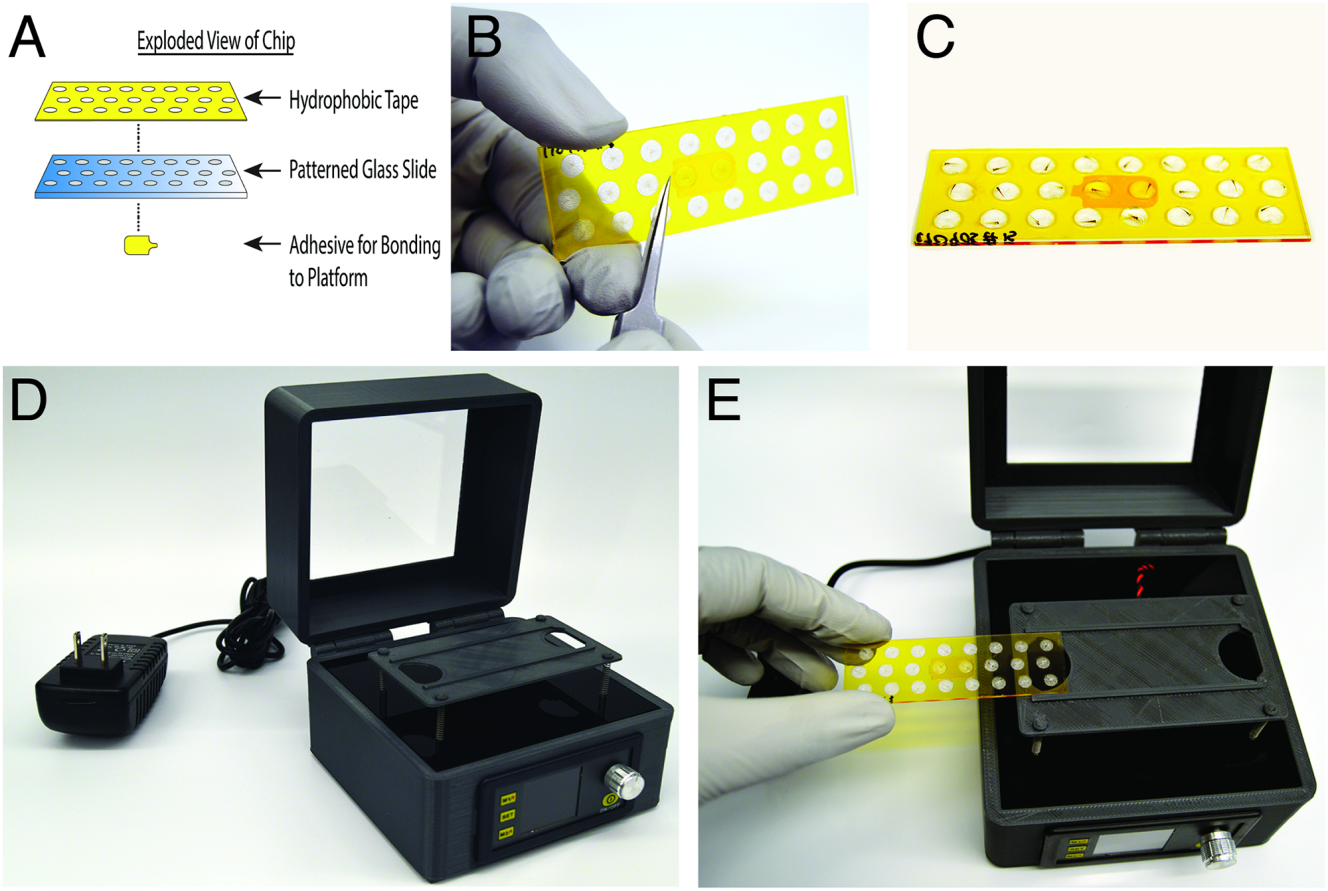
Views of glass slides (chips) for genotoyping. A) Schematic of glass slide, hydrophobic tape, and well design. B) 24 channel chip, top view; C) 24 channel chip loaded with 72 hpf zebrafish embryos; D) Base unit; E) Base unit with loading of 24 channel chip.

Extraction chips were made from standard glass microscope slides. Circular roughened areas were mechanically etched into the top surface of the glass slide. A polyimide tape was cut to the glass slide dimensions; circular holes were cut in the same configuration as the roughened areas made by the laser. The polyimide tape was aligned and attached to the glass surface creating open but shallow chambers wherein the roughened glass areas are centered in the through holes made in the polyimide tape. A completed 24 chamber chip is shown in (Fig 1A–1C). Chips having 16, 24, or 48 chambers were made.

### Device construction

The base unit operates the disposable chip. The platform holding the chip is agitated at a specific frequency which generates an abrasive environment in each chamber on the chip. The platform is suspended on springs that permit planar movement. The base unit (Fig 1D and 1E) consists of a 3-D printed (LulzBot TAZ6) device housing which contains a power supply (Uctronics U5168), a chip-holding platform with a mounted 12mm (3 V) vibration motor (Precision Microdrive 312–108) on its undersurface, the platform raised on four springs, and an evaporation-limiting cover to place over the slide.

### Device operation protocol

Embryos or larvae are manually loaded into the chip wells using a standard pipette with cut-off tips in 10–14 μL of E3 medium or water. This process takes roughly two minutes for 24 embryos. Next, the chip is loaded onto the platform of the base unit; the evaporation cover is placed over the chip; and the vibration motor is powered by supplying 1.4 volts to the motor for 7 to 10 minutes. Then, a standard pipette was used to collect 10 μL of fluid from each chamber. This 10 μL sample contains genetic material from the embryo. Care is taken to avoid touching the embryo with the pipette tip. Immediately following the removal of the fluid, the embryo is transferred from the chip using a standard 2 mL disposable transfer pipette with E3, to a 96-well plate or Eppendorf tube. Fluid levels for each embryo were maintained at > 300 μL.

## Results

### Designs and testing

Our goal was to make a device that could reliably obtain DNA samples from live zebrafish animals. We pursued two alternative approaches based on our initial publication [8]: a device collecting genetic material from the chorionic fluid; or a device that physically obtains genetic material from the embryo. We found that while a chorionic fluid device was able to obtain genetic material, that it had variable and inconsistent sensitivity that ranged as low as 0% but was typically 30%. Further, it required a large number of PCR cycles, typically >50, which introduced significant risk for contamination and false positives.

Concurrent with testing devices based on chorionic fluid analyses, we also tested genetic material extraction devices based on physical dislocation of cells from the embryo. The most direct approach was to modify fin clipping for a microfluidic chip. We tested multiple designs, but they required semi-complex feedback systems. Further, the microfluidic chip components were expensive to incorporate into a disposable chip; and a reusable chip would introduce problems with potential carry-over that could lead to contamination. We felt that a disposable chip would be optimal to prevent DNA contamination. Because of the significantly higher cost and technical obstacles we did not pursue microfluidic fin clipping after testing several devices.

We discovered that a micro-abrasion technique could generate the required genetic material with negligible impact on embryo survival. We tested multiple alternative strategies of the micro-abrasion technique. These included various combinations of roughened surfaces, chip materials, and methods of agitation. Sonication, even with low speeds or short times, was overly destructive to the embryos.

Other designs showed more promise. One design utilized a roughened glass surface upon which a polydimethylsiloxane (PDMS) channel was bonded. The embryo was then shuttled back and forth across the surface in a minimal fluid volume generating the genetic material. Another design utilized a cylindrical chamber of 20 μL molded in PDMS and bonded to a roughed pattern on a glass slide. The chip was loaded onto a modified commercial shaker plate which orbited in a 3 mm diameter circular planar motion at a specified rpm.

The final design (Fig 1), termed the Zebrafish Embryo Genotyper (ZEG) included two significant changes over prior designs. First, the PDMS chamber component was replaced with hydrophobic tape creating an open top droplet system. Loading embryos became simpler and less stressful on the embryo; and the chip cost was reduced by a factor of ten. The second change was moving from the orbital shaker plate to a coin vibration motor as a means of chip agitation. We determined that the improved abrasion environment together with higher frequency agitation led to improved survival.

### Testing of ZEG device

**Fig 2:**
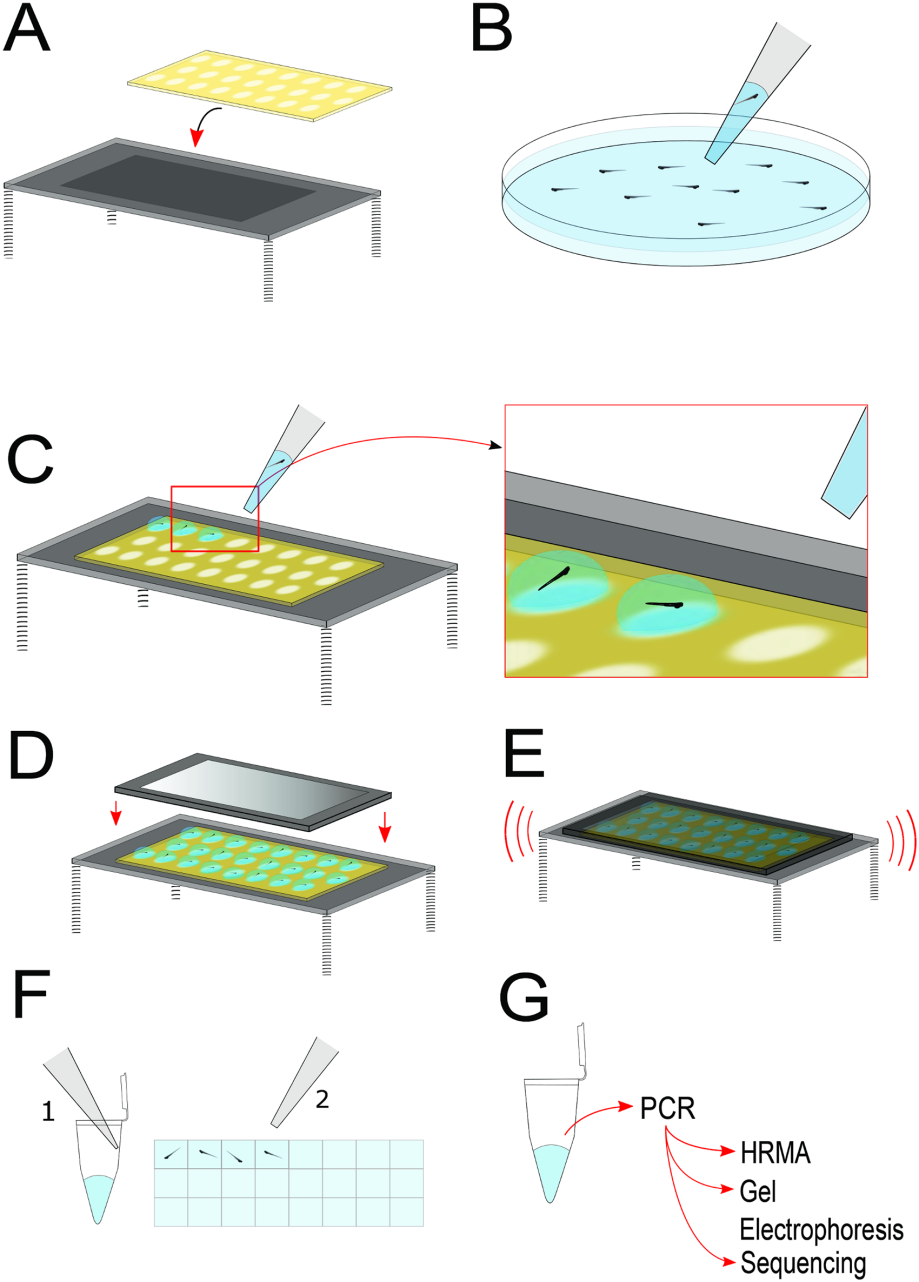
Flow diagram. Flow diagram for genetic material extraction using the ZEG.

The protocol for ZEG use is as follows (Fig 2). First, a chip is manually loaded using a standard pipette and custom tip (in which the end of the tip is cut off to widen the bore) with each embryo in 11 μL E3 buffer or water. This process takes roughly two minutes for 24 embryos. Next, the chip is loaded into the base unit and vibrated for 10 minutes. A 12 mm footprint coin vibration motor operating at 1.4 volts was used. Then, a standard micropipette was used to collect 10 μL of fluid from each chamber. Immediately following the removal of the fluid sample, the embryo is transferred from the chip using a transfer pipette containing E3 to a “holding” area (either a 96-well plate or an individual Eppendorf tube) to await the genotyping results. Fluid levels for each embryo were maintained at approximately 300 μL in the holding plate or tube.

We compared survival, PCR sensitivity, and time to completion, using two different micro-abrasion chip and device designs (Table 1). We found that the PDMS chamber device had a sensitivity of only 51%; whereas the hydrophobic tape device had both sensitivity and survival greater than 90%. The loading and unloading of the fish in the PDMS cylindrical chamber caused mechanical damage to the embryos and reduced survival.

**Table 1:** Results comparing chip and device designs. Testing results comparing two different micro-abrasion chip and device designs.

Test Description	Sensitivity	Survival	n (embryos)
Chip—20uL PDMS Chamber on Roughened Glass; Processed on Shaker Plate for 10 min at 100rpm;	51%	81%	96
Chip—Hydrophobic layer on Roughened Glass; Processed on Coin Vibration Motor System for 10 min	94%	94%	>200

**Fig 3:**
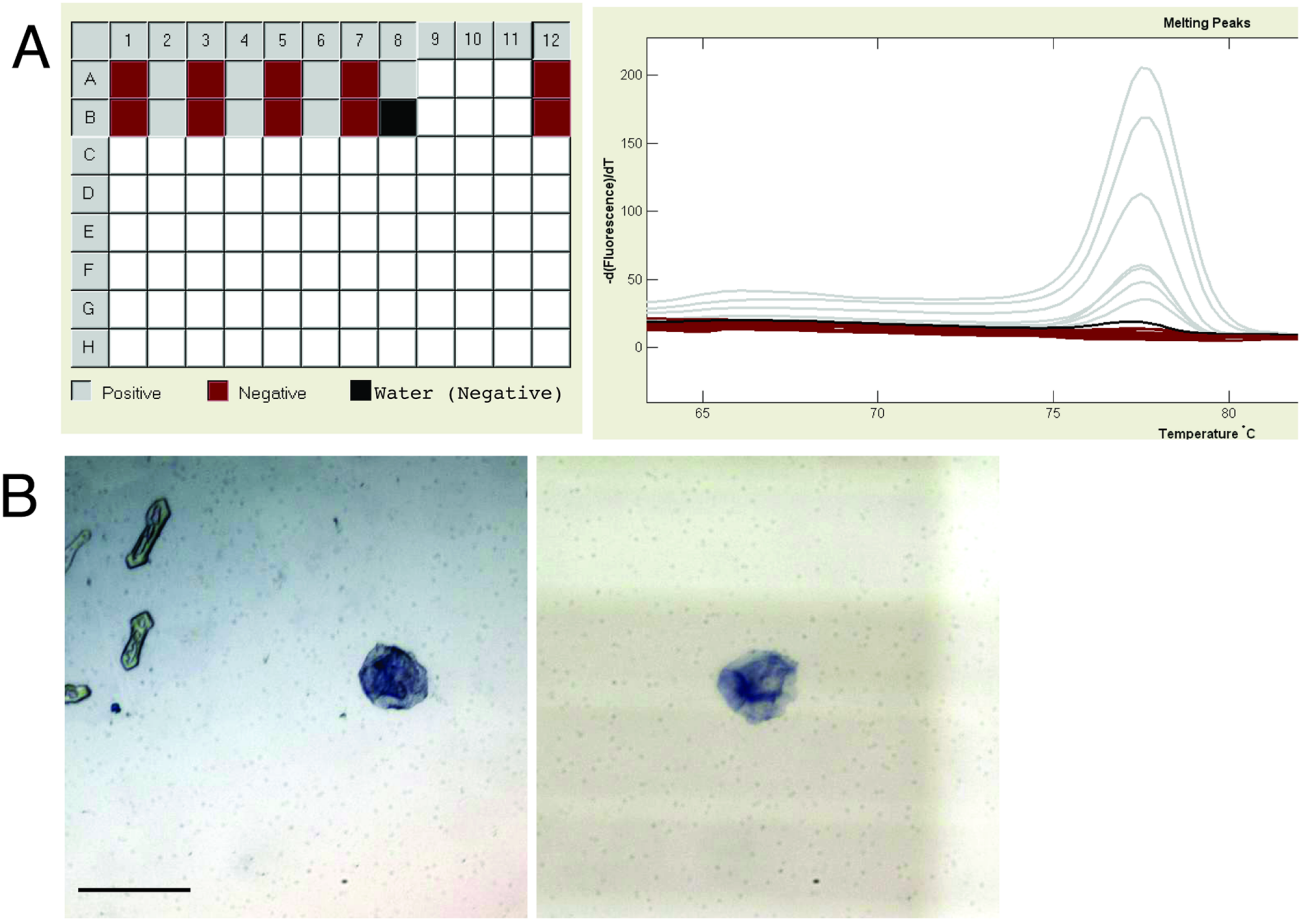
Testing of ZEG parameters. A) Evaluation for cross-contamination. Neighboring wells on a 24 channel chip were loaded either with an embryo or with a control (water blank); extraction was performed, followed by PCR and then HRMA. No signal was obtained in the water blank controls. Note, well B8 was designated in the software as a negative control and was therefore marked as a “Water (Negative)” with a block box; the maroon boxes labeled “Negative” were scored by the software algorithm as being negative/not amplifying. B) Representative images of cellular material collected following ZEG extraction; trypan blue staining. Scale bar 5 μm.

In addition, we also tested our chip design to evaluate for possible cross contamination between adjacent wells. Embryos were loaded in alternate wells on a single chip, and the remaining wells had 10 μl of water. Extraction was carried out and samples were collected. All embryos were successfully genotyped and no cross contamination was observed from the water-only wells (Fig 3A).

### Analyzing cellular material from the ZEG

To analyze the material obtained from the ZEG, we performed two tests. First, we manually visualized the samples following collection. We found that each 10 μL of sample from a single zebrafish embryo had on average 21 cells (range 2–50; standard deviation 16; standard error of the mean 3.7; n = 19 embryos), counted using a hemocytometer with trypan blue staining (Fig 3B). Almost all of the cells appeared to have been disrupted, suggesting that genetic material (e.g. DNA) present in the solution served as the template for subsequent PCR.

Second, we performed quantitative PCR of chip samples to determine how much DNA was obtained, which can be extrapolated to the number of genomes and thus number of cells. We tested 48 embryos, from two chips, with two different PCR assays. We found that each well had on average 34.3 pg of genomic DNA (range, 2.0–28.9; SD 18.6, SEM 2.7), or about 9.5 diploid zebrafish genomes (range, 1.4–20.0; SD 5.2, SEM 0.75). We think that the DNA determination by PCR is more reliable for accurate quantification, because the trypan blue determination of cells could be influenced by fragmentation of cells, making the counting not reflective (that is, likely overestimating).

These experiments demonstrate that first, that there is a source of DNA present (from cells) that provides the template for the PCR; and second, provides an estimate for the number of cells/genomes so that users can tailor their experiments (for example, if they have a PCR that requires larger numbers of cells to be reliable).

### Testing genetic material from the ZEG

**Fig 4:**
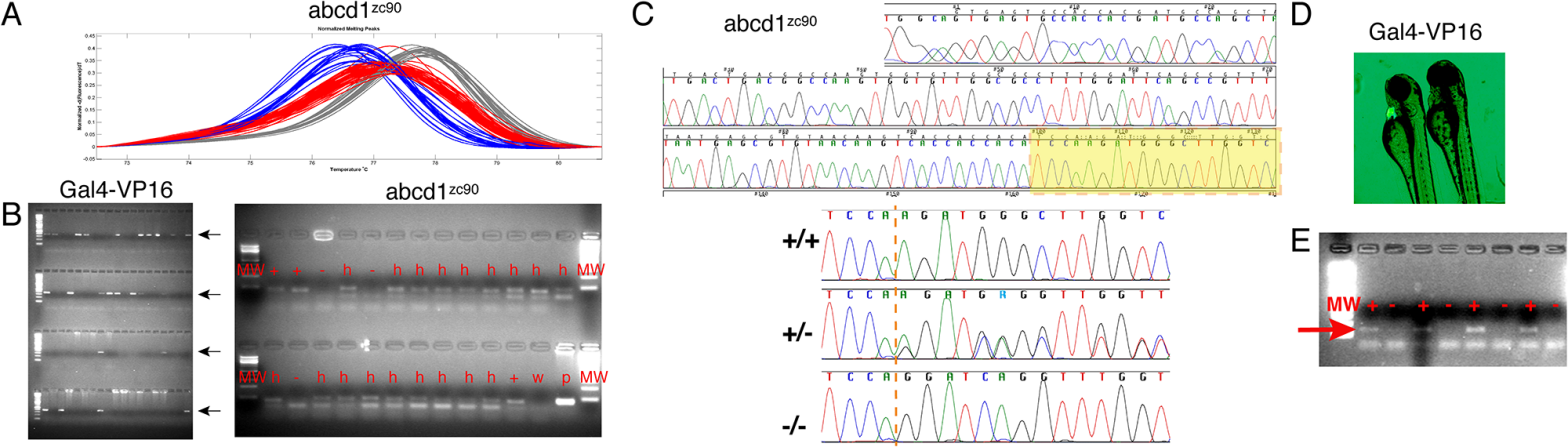
Multi-modal analysis of genetic material collected from ZEG. A) HRMA analysis of embryos carrying a nucleotide point mutation in the *abcd1* gene. Red curves, wild-type; blue curves, heterozygous mutant; gray curves, homozygous mutant. X-axis, melt temperature (°C); y-axis, normalized change in fluorescence with temperature. B) Agarose gel electrophoresis: left, PCR for Gal4-VP16 in 74 embryos; positive results are easily scored (arrows). 2% agarose gel, four rows of lanes, 20 wells/lane, molecular weight marker at far left, final two wells in upper right are negative control and positive control. Right, PCR for *abcd1*^*zc90*^; scoring is indicated above each well; MW, molecular weight; +, wild-type; -, homozygous mutant; h, heterozygous; w, water control; p, positive control. C) Chromatogram sequence results from sequencing PCR products of *abcd1*^*zc90*^ genotyping. Example of entire amplicon sequence read shown; highlighted area shown below in higher resolution for three different genotypes (wild-type, heterozygous, and homozygous). Dotted orange line indicates start of 12-bp mutant insertion. D) 72 hpf larvae; left larva is transgenic and GFP expression is visible in the heart. E) Gal4 amplicons (red arrow) from alternating GFP+ and GFP- larvae. Gal4 amplicon is seen in every lane in which the larvae was scored positive by visible presence of GFP+ heart seen under fluorescence microscope, and no amplicon in GFP- larvae. MW, molecular weight marker.

Following collection of the genetic material, we performed PCR. We then tested three different forms of analysis. First, we tested the ability of the ZEG and subsequent PCR to reliably detect and differentiate three different alleles of a splice-site nucleotide change mutation in the gene *abcd1* [11]. Following ZEG extraction, PCR, and HRMA, we were able to reliably distinguish wild-type, homozygous mutant, and heterozygous embryonic zebrafish (Fig 4A). The HRMA plot shows melting peaks for embryos from a cross of two heterozygous parents (*abcd1*^*sa509*^) processed simultaneously on a single chip by a single user.

We then examined whether the ZEG could obtain sufficient DNA for PCR to be analyzed by agarose gel electrophoresis. We did this because agarose gel electrophoresis is considered less sensitive than HRMA; also, HRMA is less commonly available. First, we again genotyped the *abcd1* locus, for the allele *zc90* (Fig 4B), and showed that we could detect and differentiate the genotypes on agarose gel electrophoresis. Second, we demonstrated that we could detect a transgene. We genotyped the offspring from crosses of three different mosaic transgene founder adult zebrafish to wild-types, a total of 74 embryos. The mosaic adults, each of which carried a Gal4-VP16 transgene, had been generated by Tol2-based transgenesis, and we had previously determined that they were mosaic founders by sacrificing and genotyping their offspring. We found that genetic material amplified from the ZEG could be visualized and used to genotype with agarose gel electrophoresis (Fig 4B). Further, all of the embryos survived the genotyping, and we were able to raise only the Gal4-VP16 transgenic offspring to adulthood. This approach saved effort and cost by raising only the desired genotype, and by-passing the requirement for fin-clip genotyping of the adults.

We also tested whether the amplified DNA from the ZEG was of sufficient quality for sequencing. We genotyped embryos from a cross of *abcd1*^*zc90*^ heterozygous adults, carrying a 12-bp insertion. Following extraction, PCR, and sequencing, we were able to obtain more than 260 bp of readable sequence (from a 294 bp PCR amplicon) and could distinguish wild-type, heterozygous, and homozygous mutant embryo sequences (Fig 4C).

Finally, we tested whether embryos were being correctly genotyped. For this experiment we used transgenic animals crossed to wild-type animals. Transgenic embryos carried a visible marker, GFP expressed under the control of a cardiac promoter, in the line Tg(*myl7*:*EGFP; foxP2-enhancerA*.*2*:*Gal4-VP16*_*413-470*_)^zc72^ [10]. In this line the construct carrying the Gal4-VP16 also carries a cardiac promotor driving GFP expression, and thus any cardiac GFP expression indicates presence of the entire transgene [13]. GFP-negative, wild-type embryos did not have PCR amplification of the Gal4 amplicon; but all GFP-positive embryos carrying the Gal4 transgene successfully amplified (Fig 4D and 4E).

### Survival, development, and behavior after chip use

An important consideration is whether the chip and extraction affect zebrafish survival, development, or subsequent behavior. In an experiment to evaluate morphology and long-term survival, after genotyping with the ZEG survival was 100% (n = 96 embryos) when followed through 7 dpf; and there were no differences in gross body morphology. Further, animals that were raised to adulthood (> 3 months) had no apparent effects on long-term growth or development and were fertile.

**Fig 5:**
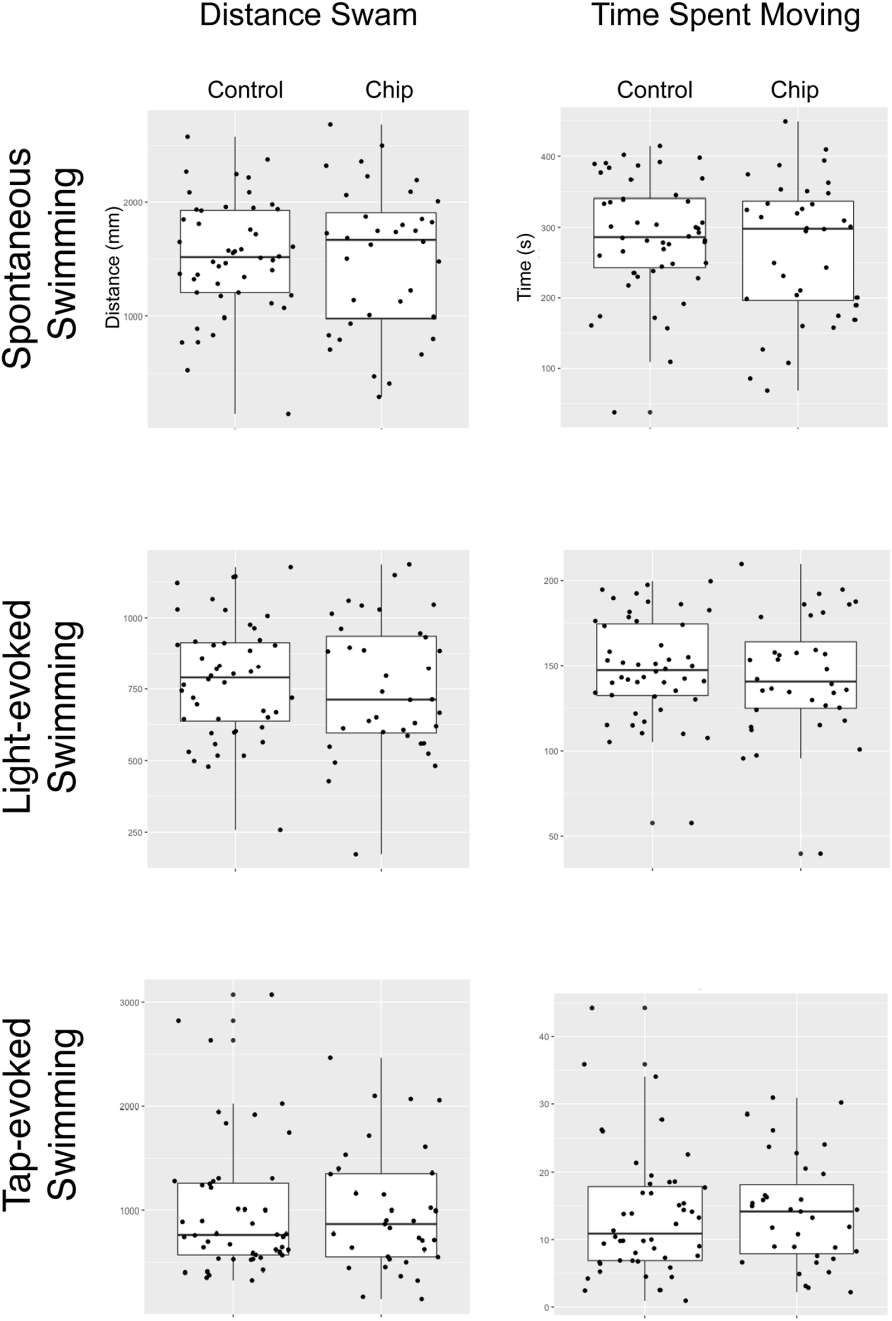
Behavior results following ZEG genotyping. Behavior results for 7 dpf larvae, comparing embryos genotyped on the ZEG to controls; there were no statistically significant differences. Box-plot analyses.

We tested behavior at 7 dpf following chip genotyping at 3 dpf. We performed spontaneous, light-evoked, and tap-evoked swimming behavior, and found no differences between chip-run larvae (n = 36) and control larvae (n = 48) from the same clutch in total distance swam (*p* = .88, .53, .64 respectively; Student’s *t*-test) or time spent moving (p = .38, .52, .78 respectively) (Fig 5).

## Discussion

We report the development and optimization of an automated device that can be used for live, high throughput genetic material extraction for genotyping of zebrafish embryos. The ZEG, or Zebrafish Embryo Genotyper device, uses harmonic oscillation of embryos over a roughened glass surface to obtain genetic material that can be directly used for genotyping. The embryos are unharmed, with no apparent adverse effects on survival, morphology, or behavior, and can be used for subsequent experiments or raised to adulthood. We have tested the ZEG for embryos older than 48 hpf that are out of their chorions; and up through age ~96 hpf larvae. The ZEG provides cells and DNA that can be used for PCR-based applications including gel electrophoresis, sequencing, and HRMA. Potentially the cells could also provide RNA or protein for analysis, although the quantities will be low and will likely be primarily epidermal in origin.

The ZEG system solves three significant issues. First and most elementarily, the system can genotype live embryos and larvae, a process necessary for mutant screens, maintenance of zebrafish stocks, testing and analysis of biochemical pathways from mutants, and for use in drug or behavioral testing. Second, the system allows researchers to identify and raise mutants of interest at an early stage of zebrafish development rather than having to wait until maturity at age two to three months. Third, this system can be used to identify homozygous, heterozygous, and wild-type embryos for a given genotype or mutation, allowing characterization of embryonic phenotypes and responses while the animals are alive. The system offers savings of time, effort, and money. Most significantly, the system offers the potential to generate and screen zebrafish mutants rapidly.

The ZEG is the only automated approach for live embryo and larval genotyping, and its speed and efficacy are appealing compared to current manual methods of genetic material collection from zebrafish embryos. The device is effective in genetic material extraction and downstream testing from a variety of genotypes and for different means of analysis. Our current estimate is that the cost of a single chip is approximately (U.S.)$10, which is economical for most labs. The PCR conditions for amplification of material from the ZEG are standard, and only use 30 cycles.

Current limitations of the ZEG are that the system is not fully automated and in particular that it requires manual loading and unloading. The manual loading/unloading introduces the potential for errors in tracking animals/results. Also, the manual loading/unloading appears to be a factor in reducing survival. We observed improved metrics in ZEG use, such as increased survival and improved sensitivity, as users become more familiar with loading/unloading of embryos. For future applications the ZEG could be further streamlined by integrating the chip such that the collected fluid could be analyzed directly, by performing the PCR on the chip. Finally, the current system has been tested and optimized for zebrafish embryos and larvae that are no longer in their chorions and are older than 48 hpf, and that are less than 96 hpf. Other improvements would target expanding the developmental age of embryos and larvae that the device could genotype.

It is likely that the ZEG will also be usable for other aquatic vertebrate species, such as Medaka or trout, but optimization of parameters may be necessary. Also, we expect that younger zebrafish, still in the chorions, could be analyzed by additional manipulations of the device, chip, and collection parameters. We think that the ability to rapidly and easily genotype large numbers of zebrafish embryos while keeping them alive will serve as an enabling technology for a wide variety of future applications.
